# Coinfection with *Trypanosoma evansi* and *Hepatozoon felis* in a free-ranging jaguar (*Panthera onca*) in the Pantanal biome, Brazil

**DOI:** 10.1590/S1984-29612026033

**Published:** 2026-07-20

**Authors:** Brenda Maria Emanuela Silva Reis, Vithoria Eduarda Barboza Silva, Jordana Toqueto, Paloma Gabrieli da Silva, Stephanie Carrelo de Lima, Alda Izabel de Souza, Carlos Alberto do Nascimento Ramos

**Affiliations:** 1 Universidade Federal de Mato Grosso do Sul – UFMS, Faculdade de Medicina Veterinária e Zootecnia – FAMEZ, Departamento de Patologia Clínica Veterinária, Campo Grande, MS, Brasil; 2 Universidade Federal de Mato Grosso do Sul – UFMS, Faculdade de Medicina Veterinária e Zootecnia – FAMEZ, Departamento de Medicina Veterinária Preventiva, Campo Grande, MS, Brasil; 3 Instituto de Meio Ambiente de Mato Grosso do Sul – IMASUL, Centro de Reabilitação de Animais Silvestres – CRAS, Campo Grande, MS, Brasil

**Keywords:** Hemoparasites, wildlife, PCR diagnosis, conservation, neotropical carnivores, Hemoparasitos, fauna silvestre, diagnóstico por PCR, conservação, carnívoros neotropicais

## Abstract

Coinfection with *Trypanosoma evansi* and *Hepatozoon felis* represents a rare occurrence in wild felids, with no previous reports in *Panthera onca*. A free-ranging adult male jaguar weighing approximately 70 kg was examined after being rescued from wildfires in the Brazilian Pantanal (Corumbá, Mato Grosso do Sul). The initial hematological evaluation (D0) revealed mild anemia (PCV: 27.7%; reference: 30–45%), with *Trypanosoma* spp. trypomastigotes forms detected by direct microscopy. Treatment with diminazene aceturate (3.5 mg/kg, IM) was instituted on D1. On D5, microscopy revealed *Hepatozoon felis* gamonts within leukocytes, characterizing coinfection. Molecular analysis by PCR confirmed the presence of both agents in the D0 sample, with amplification products of approximately 600 bp (*Hepatozoon* spp*.*) and 196 bp (*T. evansi*), validating the microscopic diagnosis. Despite treatment and the absence of microscopically detectable parasitemia on D12, anemia persisted (PCV: 21.7%). The animal progressed to death on D19 during sedation for wound management. At necropsy, severe pulmonary involvement (bronchiolitis, alveolitis, and endogenous lipid pneumonia) compatible with smoke inhalation injury was observed. These findings highlight the importance of integrating hemoparasite surveillance into rehabilitation and conservation protocols for threatened felids in fire-affected ecosystems.

## Resumo

Coinfecção por *Trypanosoma evansi* e *Hepatozoon felis* representa ocorrência rara em felídeos silvestres, sem relatos prévios em *Panthera onca*. Foi examinado um macho adulto de onça-pintada de vida livre pesando aproximadamente 70 kg, resgatado dos incêndios florestais no Pantanal brasileiro (Corumbá, Mato Grosso do Sul). A avaliação hematológica inicial (D0) revelou anemia discreta (VG: 27,7%; referência: 30-45%), com formas tripomastigotas de *Trypanosoma* spp. detectadas por microscopia direta. Tratamento com aceturato de diminazeno (3,5 mg/kg, IM) foi instituído em D1. Em D5, a microscopia revelou gamontes de *Hepatozoon felis* no interior de leucócitos, caracterizando coinfecção. Análise molecular por PCR confirmou a presença de ambos os agentes na amostra D0, com produtos de amplificação de aproximadamente 600 pb (*Hepatozoon* spp*.*) e 196 pb (*T. evansi*), validando o diagnóstico microscópico. Apesar do tratamento e da negativação da parasitemia detectável em D12, a anemia persistiu (VG: 21,7%). O animal evoluiu para óbito em D19 durante sedação para manejo de feridas. À necropsia, observou-se comprometimento pulmonar severo (bronquiolite, alveolite e pneumonia lipídica endógena) compatível com lesão por inalação de fumaça. Esses achados reforçam a importância de integrar a vigilância de hemoparasitoses nos protocolos de reabilitação e conservação de felídeos ameaçados em ecossistemas sujeitos a incêndios.

Wild carnivores play a crucial role as reservoir and maintenance hosts for a wide range of pathogens, being capable of harboring and transmitting zoonotic agents and those of veterinary importance. However, health investigations in these animals face logistical constraints and diagnostic challenges, which may hinder the detection and understanding of the epidemiology of these infections (Rojas et al., 2024; Deem et al., 2001). The jaguar (*Panthera onca*), the largest felid in the Americas, is classified as “Near Threatened” according to Quigley et al. (2017), reinforcing the relevance of monitoring its diseases, particularly to support control and conservation actions for the species.

Among hemoparasites of relevance in wild mammals, *Trypanosoma evansi*, the etiological agent of “surra” or “mal das cadeiras,” stands out. This trypanosomiasis, widely distributed in tropical regions, primarily affects equines and dogs and is mechanically transmitted by hematophagous dipterans (Desquesnes et al., 2013; Radwanska et al., 2018). Another important group comprises protozoa of the genus *Hepatozoon*, whose infection in carnivores occurs primarily through the ingestion of arthropod vectors containing mature oocysts. Both agents may present with variable clinical signs, ranging from subclinical infections to severe systemic conditions, depending on host immunity and parasite burden (Baneth & Allen, 2022).

Although isolated cases of infection by *Trypanosoma evansi* (Fagundes-Moreira et al., 2024) and *Hepatozoon* spp. (Furtado et al., 2017; Alves et al., 2025) have already been described in jaguars, coinfection by these agents in felids is considered rare and, to the best of current knowledge, had not been described in jaguars (*Panthera onca*). The simultaneous occurrence of these agents raises concern, as parasitic association may potentiate the inflammatory response and exacerbate hematological disorders through pathogenic synergism. This phenomenon has been observed in coinfected domestic animals (Bastos et al., 2021). Such a condition compromises the physiological reserve of individuals already subjected to environmental and anthropogenic stressors. In addition, habitat sharing with domestic animals favors the bidirectional flow of pathogens between jaguars and domestic species (Herrera et al., 2011), making monitoring essential from a One Health perspective.

Thus, the present study aims to report a novel case of natural coinfection by *T. evansi* and *Hepatozoon felis* in a jaguar in the Brazilian Pantanal, diagnosed through the combination of parasitological and molecular methods.

An adult male free-ranging jaguar (*Panthera onca*), weighing approximately 70 kg, was rescued in the Brazilian Pantanal, Passo do Lontra – Nhecolândia, Corumbá, Mato Grosso do Sul, Brazil (19°31′21.5″S, 57°04′28.8″W), in August 2024 and referred to the Wildlife Rehabilitation Center (CRAS/IMASUL) in Campo Grande, Mato Grosso do Sul. The animal presented burn injuries resulting from forest fires that affected the region. On the day of admission (D0), the animal was lethargic, had a body condition score of 2/5, signs of dehydration, and third-degree burns affecting all four limbs.

As part of the health screening protocol, blood samples were collected in EDTA tubes for complete blood count and hemoparasite investigation. The initial hematological evaluation revealed mild anemia (PCV: 27.7%; reference values for *Panthera onca*: >30%) (Widmer et al., 2012), with the remaining hematological parameters within reference ranges. Parasitological screening was performed using blood smear, buffy coat, thick drop, and the Woo technique (centrifugation at 12.000 rpm for 5 minutes) (Woo, 1970), allowing, at this initial stage, the visualization of forms compatible with *Trypanosoma* spp. in all diagnostic techniques employed (Figure 1A). It is noteworthy that, in this initial evaluation (D0), microscopic examination did not reveal the presence of other hemoparasites.

**Figure 1:**
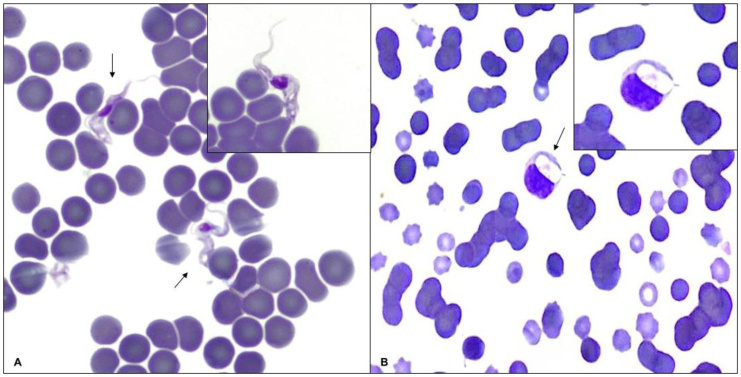
Blood smears of a free-ranging jaguar (*Panthera onca*) with coinfection by *Trypanosoma evansi* and *Hepatozoon felis* (Panoptic Rapid staining, 1000× magnification, oil immersion). (A) Trypomastigote form of *T. evansi* (arrow) among erythrocytes. Note the characteristic morphology of the parasite; (B) Gamonts of *Hepatozoon felis* (arrow) within a leukocyte. Note the intracellular structure of the parasite, confirming coinfection diagnosis on day D5 of hospitalization.

Following the initial parasitological diagnosis, specific treatment with diminazene aceturate (3.5 mg/kg, intramuscularly, single dose) was instituted on D1, associated with fluid therapy, supportive analgesia, and treatment of burn lesions. The animal was maintained under daily clinical monitoring with serial hematological evaluations.

On D5, microscopy of the blood smear stained with Panoptic Rapid revealed *Hepatozoon felis* gamonts within leukocytes (Figure 1B), characterizing coinfection. Parasitemia by *T. evansi*, assessed by blood smear stained with Panoptic Rapid on D5, revealed approximately two trypomastigotes per microscopic field (oil immersion objective, 1000×). *Hepatozoon* gamonts were observed in approximately 1% of the examined leukocytes. The remaining hematological parameters remained within reference ranges.

On D12, no circulating parasitic forms were detected in direct examinations (blood smear, thick drop, and microhematocrit). However, anemia persisted and worsened (PCV: 21.7%), with the remaining hematological parameters remaining within reference limits, except for mild reactive thrombocytosis.

The animal progressed to death on D19 during a sedation procedure for wound management. At necropsy, severe pulmonary involvement (bronchiolitis, alveolitis, and endogenous lipid pneumonia) compatible with smoke inhalation injury was observed, with no macroscopic evidence of visceral damage associated with hemoparasites.

For taxonomic confirmation of the agents and validation of the diagnosis, aliquots of whole blood collected at admission (D0) and stored frozen at −80 °C until processing were used. Genomic DNA was extracted as described by Araújo et al. (2009). DNA sample integrity and concentration were assessed by electrophoresis on 1.5% agarose gel and spectrophotometry (260/280 nm) using a NanoDrop One C (Thermo Fisher Scientific).

Detection of *Hepatozoon* spp. was performed by PCR targeting the 18S rRNA gene, as described by Vargas-Hernandez et al. (2012), using the primers HepF (5′-ATACATGAGCAAAATCTCAAC-3′) and HepR (5′-CTTATTATTCCATGCTGCAG-3′). For detection of *Trypanosoma evansi*, the protocol for amplification of the invariant surface glycoprotein (ISG) gene was followed, as described by Kumar et al. (2016), using the primers ISG196-F (5′-AAAGCCACCGAAGATGCAGA-3′) and ISG196-R (5′-TTGTCCCAATCCAGCCACTC-3′), with an expected amplicon size of 196 bp.

PCR amplification was positive for both agents in the D0 sample, supporting the molecular diagnosis of coinfection by *Hepatozoon* spp. and *T. evansi.* PCR products were visualized on 1.5% agarose gel stained with GelRed® Nucleic Acid Gel Stain (Biotium, San Francisco, USA), showing an approximately 600-bp band for *Hepatozoon* spp. and a band corresponding to the expected ISG amplicon for *T. evansi*. Subsequently, the sequence obtained from the *Hepatozoon* amplicon showed 100% identity with *Hepatozoon felis* sequences available in GenBank-NCBI. The sequence was deposited under accession number PZ291831. The amplicon obtained for *T. evansi* detection was not sequenced.

This study documents the first molecular record of coinfection by *Trypanosoma evansi* and *Hepatozoon felis* in *Panthera onca* in the Pantanal. Unlike a previous report in sympatric carnivores describing asymptomatic infections (André et al., 2010), the animal presented severe anemia (PCV 21.7%) and apathy. The clinical severity contrasts with the stable endemicity of these agents in the region (Alves et al., 2025) and suggests that coinfection, associated with stress and burn-related trauma, disrupted the parasite–host equilibrium. Previous reports have documented *Hepatozoon* infection in other neotropical felids in the Pantanal, including ocelots (*Leopardus pardalis*) and southern tiger cats (*Leopardus tigrinus*) (Braz & Umeda, 2015; Metzger et al., 2008), generally presenting an asymptomatic course. Recently, Fagundes-Moreira et al. (2024) reported the detection of *Trypanosoma evansi* in free-ranging jaguars in the Pantanal. The absence of severe clinical manifestations in these reports contrasts with the progression observed in the present case, reinforcing that thermal trauma and pulmonary impairment were determining factors in the observed clinical outcome.

The pathophysiology of the condition is directly related to thermal trauma and its systemic effects. Extensive burns trigger an inflammatory response, which may directly contribute to anemia through suppression of erythropoiesis and iron sequestration, in addition to hypermetabolism and immunosuppression (Jeschke et al., 2011), favoring the reactivation of latent infections. The late detection of *Hepatozoon felis* on D5, absent at admission, suggests an increase in parasitemia induced by physiological stress and pulmonary inflammation secondary to smoke inhalation. The persistence and worsening of anemia (PCV from 27.7% at D0 to 21.7% at D12) after treatment with diminazene, despite the absence of microscopically detectable parasitemia, associated with severe respiratory lesions observed at necropsy, indicate that death resulted from the synergistic interaction between parasitic coinfection, thermal trauma, and pulmonary impairment. In a study conducted in domestic felines experimentally infected with *Trypanosoma evansi*, the development of anemia was also described, associated with multiple pathophysiological mechanisms. Notably, especially under conditions of high parasitemia, erythrocyte damage may occur both due to mechanical effects resulting from parasite motility and through the interaction of surface proteins with erythrocytes, promoting their removal by the mononuclear phagocyte system, processes widely implicated in the pathogenesis of anemia in trypanosomiasis (Silva et al., 2011). In dogs coinfected with *Hepatozoon canis* and other hemoparasites, synergistic effects have been associated with more pronounced anemia and greater immunosuppression (Baneth & Allen, 2022), suggesting that parasitic coinfections may potentiate hematological impairment in wild felids with reduced physiological reserve. This vulnerability is further aggravated by anthropogenic effects such as climate change, habitat fragmentation, and capture, which alter the neuroendocrine axis and reduce immunocompetence in wildlife (Deem et al., 2001; Macedo et al., 2021).

From a diagnostic standpoint, this case highlights the value of a complementary approach combining direct parasitological methods and molecular techniques. Blood smears, thick drop, and microhematocrit (Woo, 1970) allowed the visualization of *Trypanosoma* spp. trypomastigotes from admission. The subsequent detection of *Hepatozoon felis* gamonts within leukocytes (D5) illustrates the importance of serial microscopic evaluation in rescued animals. PCR confirmed the molecular identity of both agents in the initial sample (D0), when parasitemia by *Hepatozoon felis* was insufficient for microscopic detection (Criado-Fornelio et al., 2009). This temporal discordance between microscopy and PCR highlights the superior sensitivity of molecular techniques for detecting fluctuating or low-level parasitemias (Thomas et al., 2024). The detection of *Hepatozoon* spp. in large neotropical felids has been increasingly documented (André et al., 2010; Furtado et al., 2017; De Sousa et al., 2017; Alves et al., 2025); however, coinfection with *T. evansi* in *P. onca* had not been previously reported. From a One Health perspective, the detection of coinfection in an apex predator underscores the role of large felids as epidemiological sentinels in livestock–wildlife interface areas (Macedo et al., 2021; Herrera et al., 2011).

Several limitations should be acknowledged. The report involves a single individual, precluding inferences regarding prevalence or population-level impact. Quantitative methods (qPCR) were not employed to estimate parasite load, limiting the understanding of coinfection dynamics (Thomas et al., 2024). The coexistence of extensive burns, inhalation pneumonitis, and coinfection hinders the attribution of relative contributions of each factor to the fatal outcome. Other pathogens relevant to felids were not comprehensively investigated.

This report documents the first case of natural coinfection by *T. evansi* and *Hepatozoon felis* in *P. onca*. The clinical severity observed in the context of burns illustrates how environmental stressors and parasitic coinfections can compromise the health of threatened carnivores. These findings reinforce the need to integrate hemoparasite surveillance into conservation and One Health strategies in the Pantanal (Macedo et al., 2021).

## Data Availability

The data from this study are included in the manuscript but are available from the corresponding author upon reasonable request.
